# Minimum data cleaning recommendations for infection prevention and control acute care surveillance reporting: A solution for “garbage in, garbage out”

**DOI:** 10.14745/ccdr.v51i09a03

**Published:** 2025-10-09

**Authors:** Kathryn Bush, Joelle Cayen, Christine Blaser, Blanda Chow, Jennifer Ellison, Jennifer Happe, Caroline Quach, Christian Tsang, Olivia Varsaneux, Kristen Versluys, Victoria Williams, Robyn Mitchell

**Affiliations:** 1 Interior Health Region, BC; 2 Canadian Nosocomial Infection Surveillance Program, Public Health Agency of Canada, Ottawa, ON; 3 Centre intégré universitaire de santé et de services sociaux du Nord-de-l’Île-de-Montréal, Montréal, QC; 4 Centre de formation continue, Faculté de médecine et des sciences de la santé, Université de Sherbrooke, Sherbrooke, QC; 5 Département de médecine sociale et préventive, École de santé publique, Université de Montréal, Montréal, QC; 6 Alberta Health Services, Edmonton, AB; 7 Surveillance and Applied Epidemiology Interest Group (SAEIG), IPAC Canada; 8 Department of Microbiology, Infectious Diseases and Immunology, University of Montréal, Montréal, QC; 9 CHU Sainte-Justine, Montréal, QC; 10 Sunnybrook Hospital, Toronto, ON

**Keywords:** data quality, data cleaning, IPAC surveillance, completeness, accuracy, timeliness, recommendations

## Abstract

**Background:**

Outcome surveillance is an important component of infection prevention and control (IPAC) programs to guide healthcare decisions. It is crucial that the reported data are of the highest quality. Reviewing completeness, accuracy and timeliness of the data is important to reduce data inconsistencies. However, many IPAC staff do not have training in data cleaning or data quality activities.

**Methods:**

Expert epidemiologists across Canada have created best practice guidance for data quality activities to provide sufficient detail to improve this important patient safety activity. Most of these activities are simple checks to review the accuracy of the data without requiring additional review of the patient record or linkage to other datasets.

**Results:**

Based on consensus by surveillance experts across jurisdictions, comprehensive recommendations for data quality in IPAC surveillance programs were developed to improve completeness (22%), accuracy (68%), and timeliness (10%) of the data.

**Conclusion:**

The data quality activities list may be used in Canadian IPAC surveillance activities to support or improve existing surveillance data quality activities for IPAC programs.

## Introduction

Infection prevention and control (IPAC) surveillance programs guide important healthcare decisions, including resource allocation for IPAC programs and developing and evaluating IPAC measures. Surveillance data inform the evaluation of interventions, outbreak detection to prevent further transmission, monitoring of trends for preparedness for both seasonal and emerging infections, and resource allocation including staffing and cleaning protocols. Surveillance data also provides evidence to support guidelines and policies ((1)). It is crucial that the reported data are of the highest quality, and it is, therefore, important to determine which minimum elements of IPAC data should be reviewed and to identify data quality activities that are consistent across different datasets, institutions, and jurisdictions. Data quality encompasses different domains, but those of completeness, accuracy and timeliness are the most important considerations ((2,3)). Data are complete when all eligible patients are included as surveillance cases and all variables in the surveillance data entry form are reported. Data are accurate when cases reflect the protocol case definition and when data classification decisions are correct. Data are timely when they are available and disseminated when the results are required ((2,3)).

The Canadian Nosocomial Infection Surveillance Program (CNISP) is a collaboration between the Association of Medical Microbiology and Infectious Disease Canada (AMMI) and the Public Health Agency of Canada (PHAC) to conduct standardized surveillance of antimicrobial resistance and antimicrobial resistant organisms with Canadian sentinel acute care facilities ((1)). The CNISP data quality working group consists of Canadian surveillance experts who collectively have a background of infection prevention and control, surveillance, epidemiology and clinical expertise. They are CNISP staff or are representatives of the Canadian surveillance network sites. The group has conducted data quality activities to support CNISP surveillance since 2005.

The purpose of this project was to provide data quality activities in sufficient detail to support existing IPAC surveillance data prior to analysis and reporting by Canadian IPAC staff.

## Methods

This CNISP project built upon initial work by analysts in the Alberta Health Services (AHS) IPAC program. The list was initially compiled using the data quality activities that the AHS IPAC analysts undertook for data review and validation of each CNISP surveillance initiative, including antimicrobial resistant organisms, *Clostridiodes difficile* infection, and healthcare-acquired infections of surgical sites, bloodstream, and viral respiratory pathogens. The activities focused primarily on those data elements that require the data collector to interpret clinical events or those that are prone to data entry errors (e.g., date fields).

**Figure 1:**
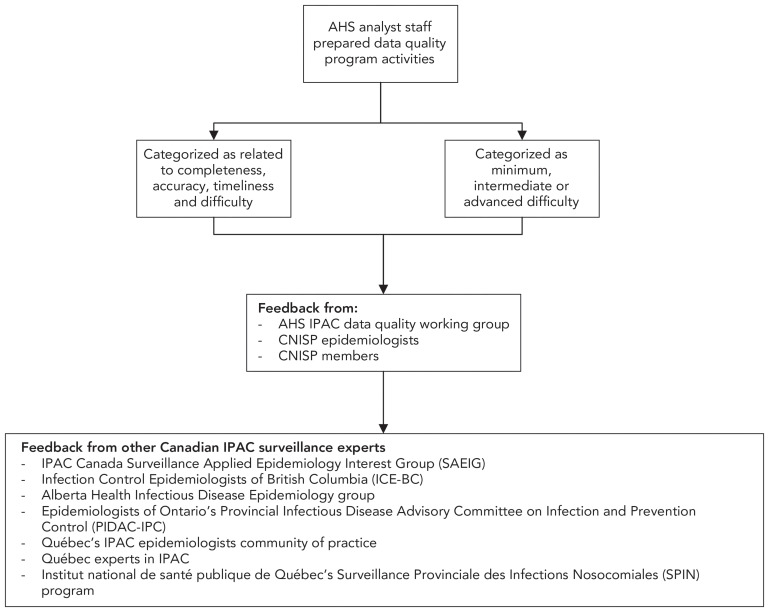
Development of the Canadian Nosocomial Infection Surveillance Program data quality activities list Abbreviations: AHS, Alberta Health Services; CNISP, Canadian Nosocomial Infection Surveillance Program; IPAC, infection prevention and control; PIDAC-IPC, Provincial Infectious Diseases Advisory Committee on Infection Prevention and Control; SAEIG, Surveillance and Applied Epidemiology Interest Group; SPIN, Surveillance provinciale des infections nosocomiales

Following that initial development, the CNISP data quality working group refined the list and then sought feedback and endorsement from surveillance experts across Canada (**Figure 1**).

The activities were described for both data elements and data processes. A data element is the smallest unit of data, and it represents a case, event or individual (e.g., lab test result, sex at birth, age, etc). A data process refers to the steps involved in collecting, managing and analyzing the data elements, transforming raw data into meaningful information.

The specific data quality activities were categorized as completeness, accuracy, or timeliness, based on the definitions of those data quality domains ((2,3)). The difficulty level reflected the ability to review the data without additional data sources or without advanced data management skills. Minimum difficulty activities were those requiring data review only without reference to other data sources. Intermediate activities required review of both the data and the patient’s record. Advanced difficulty activities required linkages with large administrative data sets (e.g., the Discharge Abstract Database) or those requiring expert physician review.

## Results

Once validated, analysis showed the data quality activities were predominantly those involving data accuracy (68%), with fewer items for completeness (22%) and timeliness (10%). Most of the data quality activities were categorized as minimum activities (71%), with four intermediate (13%) and five advanced (16%) activities (**Table 1**).

**Table 1:** Data quality domains by level of difficulty for the listed data quality activities

Data quality domain	Level of difficulty			
	Minimum^a^	Intermediate^b^	Advanced^c^	Total
Completeness	3	2	2	7 (2%)
Accuracy	17	2	2	21 (68%)
Timeliness	2	0	1	3 (10%)
Total	22 (71%)	4 (13%)	5 (16%)	31

^a^ Minimum difficulty activities: those requiring data review only without reference to other data source

^b^ Intermediate activities: requiring review of both the data and the patient’s record

^c^ Advanced difficulty activities: requiring linkages with large administrative data sets (e.g., the Discharge Abstract Database) or those requiring expert physician review

The feedback from the different Canadian epidemiology experts was given in an interview format and changes were updated. Reviewer feedback was positive, with consensus on the proposed list of data quality activities (**Table 2**). For example, one expert noted that they included these activities in their practice but appreciated suggestions for other activities they had not considered. The activities list was then approved by all the groups that were contacted. Other actions resulted from the discussion with the IPAC Canada Surveillance and Applied Epidemiology Interest Group, including their discussions with the IPAC Canada executive, resulting in endorsement from the IPAC Canada board.

**Table 2:** Canadian Nosocomial Infection Surveillance Program data quality activities list

Data quality domain	Data element, data process	Level of difficulty	Process	Additional information/activity/action
Completeness – all eligible cases are included; all data variables are reported.	Patient name, gender, healthcare identification number(s)	Minimum	Verify data entry correctness against the source of truth (clinical record).	Have options built in for linking to additional names for same person.
	Missing data in variables	Minimum	Review clinical system for data entry completeness.	Consider data validation rules within the surveillance system for mandatory data entry elements. Periodic review of missing data elements to consider whether data should continue to be collected (e.g., risk factors for carbapenemase-producing organisms (CPO) or viral respiratory infection (VRI) acquisition). Provide a report to surveillance leaders on low response data variables.
	Missing cases	Advanced	Confirm all positive, eligible specimens are considered for surveillance.	Confirm that all blood cultures taken in pre-admission (including emergency) where patients are direct transfer to inpatient care, are included since blood cultures may not be collected again during the inpatient’s admission. Consider review of laboratory data to confirm all surveillance cases were captured. Consider review of surgical procedure denominator data to confirm all included patients meet surveillance eligibility.
	Entry of outcome variables (e.g., attributable death, attributable ICU admission, attributable colectomy, other attributable adverse events)	Advanced	These adverse events are followed for 30 days after surveillance event. To ensure complete case capture, data linkages can be performed with administrative discharge data to confirm that all patients with an eligible adverse event were captured.	Discharge abstract data (DAD) contain information on discharge reason, diagnosis and unit (ICU) admission. Admission, discharge, transfer (ADT) data contain information on all patient movements during an admission.
	Denominator and numerator linkages	Intermediate	Case included which is not present in denominator.	Analyst review, then record deletion.
	“Complete” records	Minimum	After data quality checks are completed, note that the record is finalized and allow no further edits unless documented.	“Completed” records can be removed from routine data extracts for cleaning/review purposes.
Accuracy – case meets the surveillance definition; classification decisions are correct.	Duplicates	Minimum	Check for duplicate patient names and records.	Delete duplicates, allow one surveillance case per protocol period.
	Surveillance cases meet protocol case definitions and eligibility criteria (synonyms: incident case, first infection case, surveillance case)	Minimum	Check for admission to acute care site at time of detection.	Create protocol interpretation decisions for emergency inpatients, pre-admission assessments, urgent care with direct transfer to acute care.
	Case classification decision	Minimum	Check the time from admission to culture date.	Calculate “time between” to confirm classification decision with protocol (e.g., community- vs. healthcare-acquired) or case eligibility.
	Infection onset	Minimum	Check that the infection onset date meets surveillance case definition.	Use gold standard infection definitions for comparability to other surveillance systems (e.g., CNISP, National Healthcare Surveillance Network [NHSN]) for infection decisions and bloodstream and surgical site infections surveillance. Review clinical record for agreement with infection control professional (ICP) decision.
	Time from admission to culture date	Minimum	For central-line associated bloodstream infection (CLABSI) surveillance, was the central line in place for minimum time before culture positive?	Calculate “time between” for insertion date to culture date and for date of admission to ICU to culture date to determine if case meets protocol definitions.
	Valid surveillance case for multiple data entries	Minimum	Check the time between surveillance cases for healthcare-acquired infections ((4)).	Confirm the new case with protocol definitions ((4)).
	Date of birth matches date of hospital admission	Minimum	Indicates data entry error for adults.	Edit record to correct errors. Note: may be correct for newborns.
	Date of birth matches lab collection date	Minimum	Indicates data entry error.	Note: may be correct for newborns.
	Date of birth matches date of infection onset	Minimum	Indicates data entry error.	Note: may be correct for newborns.
	Date of hospital admission matches date of lab collection	Minimum	Confirm if hospital-acquired infection.	Confirm timeframe of case classification definition.
	Date of infection matches date of procedure	Minimum	For surgical site infection surveillance.	An SSI case cannot occur on the same day as the first surgical procedure.
	Formatting errors in date fields	Minimum	Data entry error, e.g., month/year.	Use yyyy/mmm/dd date format.
	Organism name (CPO, BSI, CLABSI, SSI, VRI)	Minimum	Confirm and correct pathogen names and distinguish between different strains of the same pathogen, determine case eligibility for common commensals and handle data related to multiple pathogens within a single case.	Compare data to clinical health record and lab data source. Use NHSN “common commensal” table as gold standard.
	Culture site for ARO surveillance	Minimum	Confirm if clinical specimen site is entered.	If surveillance definition requires an infection to be present, antimicrobial resistant organism screening sites (e.g., nasal screening specimens) are not allowed for surveillance cases.
	“other” responses	Minimum	Confirm that “other” is not selected if the valid response is included in a checklist/pull-down menu.	Review all free text entries to minimize use.
	Formatting errors in text data variables	Minimum	Examples: hyphens included/not included in accession numbers, variability in site or unit name (acronym, spelling errors).	Review data quality to system standard. Consider drop-down fields in data entry system for consistency.
	Valid date of death	Minimum	Do not include deaths beyond protocol timeframe.	Most protocols ask for outcome at 30 days after surveillance record date. Do not include deaths or other outcomes greater than 30 days.
	Lab data: multiple specimens collected	Intermediate	Review lab and/or surveillance records compared to the clinical record: if multiple specimens were collected from different locations, inpatient positive specimens are selected as described by the patient population of each protocol.	Create additional data entry guidance for users to allow future lab data linkages for case-finding purposes for example, if the specimens have similar levels of clinical relevance (e.g., urine and wound), the specimen collected first is selected. If there are multiple accession numbers for the same microbe from multiple specimen sites, the more clinically relevant specimen is selected. If there are multiple specimens collected at the same time, the specimen reported first is selected.
	Encounter information: facility, unit, service, bed	Intermediate	Surveillance case is entered for where case is attributed rather than where case is detected.	Review clinical record, consider standard process for entering cases where patients have had multiple transfers (i.e., last unit patient was on vs. unit where patient was on at time of lab collection).
	Infection/colonization decision	Intermediate	Send back cases to the data collectors for re-review, to confirm the NHSN infection decision.	Periodic data quality activity: especially for cases with sputum, wound and urine specimens, which have higher likelihood of organism colonization.
	Symptom fields for VRI surveillance	Intermediate	Review virus name and associated symptoms to determine surveillance case eligibility.	Pathogen can determine which symptoms are considered as surveillance case.
	Vaccination status	Advanced	Cannot indicate COVID-19 or other vaccinations prior to vaccine availability.	Need to track different vaccine availability dates and indication in each province.
Timely – surveillance data are provided at the time the data are needed.	Time from lab to data entry	Minimum	Consider data entry timeline expectations to provide timely data, e.g., data entered within 5 days of positive culture date.	Time between data entry date and culture date – provide feedback to ICPs and leaders, work to create efficiencies to allow prompt surveillance data entry.
	Time for review	Minimum	Determine data quality activity frequencies.	Review each type of data quality check with reporting timelines to determine the frequency or schedule of required data cleaning (daily, weekly, monthly).
	Time to reporting	Minimum	Determine frequency of reporting (daily, weekly, monthly, quarterly, annually).	Create data quality activities and timelines to accommodate data reporting frequency, including time for review with ICPs.
	Denominators for reporting	Advanced	Determine frequency of preliminary and final rate reporting.	Consider denominator data source and ability for timely case finding process to match stakeholder needs and data availability. Includes: patient-days, admissions, line-days, surgical procedures.

Abbreviations: ADT, admission, discharge, transfer; ARO, antibiotic-resistant organism; BSI, bloodstream infection; CLABSI, central-line associated bloodstream infection; CNISP, Canadian Nosocomial Infection Surveillance Program; CPO, carbapenemase-producing organisms; DAD, discharge abstract data; ICP, infection control professional; ICU, intensive care unit; NHSN, National Healthcare Surveillance Network; SSI, surgical site infection; VRI, viral respiratory infection

## Discussion

The purpose of this project was to provide data quality activities in sufficient detail to support existing IPAC surveillance data prior to analysis and reporting by Canadian IPAC staff. As far as we are aware, this is the first detailed list of data quality activities for staff reporting IPAC surveillance results in Canada. The collaboration between Canadian IPAC surveillance experts has been important in validating these data quality activities and sharing them with IPAC programs across the country. Other programs have recommended data quality activities, including the Centers for Disease Control National Healthcare Surveillance Safety Network (NHSN), the Canadian Institute for Health Information (CIHI), and the Australian Commission on Safety and Quality in Health Care (ACSQHC) ((3,5–7)). These experts recommend creating an overall surveillance plan to understand data sources, data validation (including administrative and laboratory data linkages to validate case-finding), and other system-level checks, but with the exception of the Australian resource, do not offer specifics.

Performing IPAC outcome surveillance is a primary accountability of any IPAC program that considers due diligence in creating complete, accurate and timely data to be an important requirement prior to reporting results. A recent publication estimated that 45% of an infection control professional’s time is directed towards surveillance activities and that manual systems may have an accuracy of only 62.5% (range: 16%–87%) ((8)). Based on this sub-optimal reported data accuracy, we aimed to develop a list to help improve the data quality of IPAC surveillance data. Although the CNISP data quality activities list is designed for CNISP surveillance, its value is that any IPAC staff member who is collecting and reporting surveillance data can use the suggested activities to improve their program’s surveillance data quality.

Although this list offers data checks for completeness, fields that are difficult to collect and are often not reported should be routinely reviewed to determine their usefulness in surveillance reporting. Identifying required data elements and reviewing incomplete data submissions can often guide data entry towards the essential data required for reporting. A minimum basic data entry set is typically created from expert consensus for the required data items that are essential for reporting, to reduce the burden of data collection and improve the quality of the data submissions ((9)). Advanced quality activities involve using other sources of data to confirm that case finding is equivalent across the surveillance system and that all potential cases are reviewed, even if missed by the original data collector.

Accurate data begins with education and supports to the data collectors regarding the protocol’s case definitions and inclusion/exclusion criteria. To evaluate the application of CNISP protocol definitions among hospitals reporting data for CNISP surveillance, the data quality working group has conducted several studies. These results show a correct response rate of 88% for bloodstream infection surveillance ((10)), 79% for COVID-19 surveillance ((11)), and 78% for *Clostridiodes difficile* infection surveillance (unpublished). Anecdotal reports have indicated that sites continue to use the survey questions when providing orientation for new staff to have discussions on correct CNISP protocol interpretation.

In the timeliness domain, there is a need for each reviewer to create a surveillance reporting schedule, so that expectations for data quality frequency checks and data entry timeliness can be set. Additional audits can be scheduled to spot-check the data quality by another reviewer as a quality control step.

The CIHI provides two additional data quality domains: relevance (the data meets the users’ current and potential needs) and accessibility (the surveillance results are easily accessed and clearly presented in a way that is understood) ((5)). These are also important domains that allow for clinical partners to understand and use the data. Discussions with clinical partners can confirm that both the infections under surveillance and the data presented are actionable, as well as endorse the surveillance definitions to capture clinically relevant cases.

For generalizability, the CNISP data quality activities list is designed for different levels of checks. Minimum checks, which any IPAC staff member can perform, include 71% (22/31) of the total activities, of which 77% (17/22) are accuracy checks. This provides flexibility in performing data quality checks to accommodate surveillance systems that are not resourced to perform intermediate or advanced level checks. One reviewer comment recognized the usefulness of IPAC surveillance analysts to support the epidemiologist in performing data quality activities, and these best practice activities can provide a business case rationale for analyst positions.

## Limitations

Any metrics of local data improvement with the use of these data quality activities relies on the state of the original reported data. Some sites may have other interventions in place—such as ongoing education initiatives to help the data collectors with more accurate protocol interpretation—which would affect that site’s overall data improvement. However, the fundamental “garbage in, garbage out” rule of data integrity applies to all data. The IPAC surveillance data have the advantage of being generated by the infection control professionals as primary data collectors, and therefore have the advantage over administrative data in the ability to improve the overall quality of the data and in avoiding the issues with administrative data, such as misleading conclusions because of the overall data inaccuracy ((12)).

Future plans for this work include dissemination of the data quality activities list and implementation of training in the CNISP network and for IPAC Canada surveillance data collectors. An evaluation of the usefulness, relevance, completeness and effectiveness of the list will follow once it has been in use for a few years, with a future version to include any suggestions for improvement.

## Conclusion

The outlined data quality activities provide a list that establishes a standard set of data quality activities to ensure complete, accurate and timely reporting of IPAC surveillance data. The activities have been reviewed by IPAC surveillance experts across Canada and validated by the AHS IPAC program, and may be used to support or improve existing surveillance data quality activities in IPAC departments. Routine application of the CNISP data quality activities list may be used to support or improve existing surveillance data quality activities in IPAC departments and increase the confidence of the users of the surveillance results.
